# Magnesium improved fruit quality by regulating photosynthetic nitrogen use efficiency, carbon–nitrogen metabolism, and anthocyanin biosynthesis in ‘Red Fuji’ apple

**DOI:** 10.3389/fpls.2023.1136179

**Published:** 2023-02-23

**Authors:** Ge Tian, Hanhan Qin, Chunling Liu, Yue Xing, Ziquan Feng, Xinxiang Xu, Jingquan Liu, Mengxue Lyu, Han Jiang, Zhanling Zhu, Yuanmao Jiang, Shunfeng Ge

**Affiliations:** State Key Laboratory of Crop Biology, College of Horticulture Science and Engineering, Shandong Agricultural University, Tai’an, Shandong, China

**Keywords:** apple, Mg, ^13^C, ^15^N, sugar, anthocyanin, gene expression

## Abstract

**Introduction:**

Both nitrogen (N) and magnesium (Mg) play important roles in biochemical and physiological processes in plants. However, the application of excessive N and insufficient Mg may be the factor leading to low nitrogen utilization rate (NUE) and fruit quality degradation in apple production.

**Methods:**

In this study, we analyzed the effects of different application rates of Mg (0, 50, 100, 150, 200 kg/ha) on the photosynthetic nitrogen use efficiency (PNUE), the accumulation and distribution of carbon (C), N metabolism, anthocyanin biosynthesis and fruit quality of the ‘Red Fuji’ apple in 2018 and 2019.

**Results:**

The results showed that the application of Mg significantly increased the ^15^NUE and increased the allocation rate of ^15^N in the leaves whereas the ^15^N allocation rate in the perennial organs and fruits was decreased. With the increase in Mg supply, the activities of N metabolism enzymes (NiR, GS, and GOGAT) were significantly promoted and the content of intermediate products in N metabolism ( ${\text{NO}}_{2}^{-}$ , ${\text{NH}}_{4}^{+}$ , and free amino acid) was significantly decreased. Furthermore, an appropriate rate of Mg significantly promoted the net photosynthetic rate (P_n_) and photosynthetic nitrogen use efficiency (PNUE), enhanced the enzyme activities of C metabolism (SS, SPS, S6PDH), and increased the contents of sorbitol and sucrose in leaves. In addition, Mg upregulated the gene expression of sugar transporters (*MdSOT1*, *MdSOT3*, *MdSUT1*, and *MdSUT4*) in fruit stalk and fruit fresh; ^13^C isotope tracer technology also showed that Mg significantly increased the ^13^C allocation in the fruits. Mg also significantly increased the expression of anthocyanin biosynthesis genes (*MdCHS* and *MdF3H*) and transcription factors (*MdMYB1* and *MdbZIP44*) and the content of anthocyanin in apple peel.

**Conclusion:**

The comprehensive analysis showed that the appropriate application of Mg (150 kg/ha) promoted PNUE, C–N metabolism, and anthocyanin biosynthesis in apple trees.

## Introduction

Mg is a vital macronutrient for plant growth and plays many important roles in biochemical and physiological processes in plants, including the synthesis of chlorophyll, nucleic acids, and proteins; the formation and utilization of ATP; and enzyme activation and participation in photosynthesis (Shaul, 2002; Marschner, 2012; Cakmak, 2013; Verbruggen and Hermans, 2013). An appropriate application of Mg can promote plant growth, increase photosynthetic efficiency (Tian et al., 2021), improve crop yield and quality (Orlovius and Mchoul, 2015; Yang et al., 2012), and also significantly promote the absorption of N, P, K, Ca, and other nutrients (Ding et al., 2012). However, the application of Mg is neglected by China’s apple industry, and the application rate of Mg fertilizer is almost negligible. The lack of Mg may not only inhibit plant growth and reduce the utilization rate of nutrients but also decrease fruit yield and quality. Therefore, Mg cannot be ignored in the management of apple nutrient resources.

Mg plays a key role in maintaining N homeostasis in plants (Peng et al., 2020b). Both Mg and N are components of chlorophyll, protein, and various structural substances in plants, playing an important role in photosynthesis and the formation of crop quality (Bloom and Arnold, 2015; Chen et al., 2018). Both N absorption and assimilation depend on H^+^-ATP for energy, whereas ATP synthesis depends heavily on the participation of Mg (Tang et al., 2012b; Tian et al., 2021). Studies have shown that Mg can promote N absorption and assimilation of crops. Ding et al. (2006) showed that there was a unimodal curve relationship between the activities of nitrate reductase (NR) and glutamine synthase (GS) and Mg content in rice leaves. Marschner (2012) found that Mg application was beneficial to leaf protein synthesis. Ding et al. (2012) found that the absorption of cabbage N and Mg can be significantly increased by the appropriate proportion of N and Mg. Grzebisz (2013) found that the application of Mg can achieve higher N utilization under the condition of low N application. In addition, Mg increased the nitrogen utilization rate (NUE) of maize, winter wheat, and sugar beet (Szulc et al., 2008; Potarzycki, 2011; Zhang et al., 2020b; Poglodzinski et al., 2021). It is seen that scientific application of Mg may have great potential to improve the NUE in the apple industry.

Scientific and effective supply of Mg not only is beneficial to N metabolism but also has a significant effect on the improvement of crop yield and quality. It had been reported in corn (Jezek et al., 2015), sunflower (Ertiftik and Zengin, 2016), radish (Kleiber et al., 2012), citrus (Tang et al., 2012a), potato (Orlovius and Mchoul, 2015), and other crops. Mg deficiency restricts the loading of carbohydrates in the phloem, resulting in obstructed transport of photosynthates to sink organs (Ishfaq et al., 2022). The accumulated carbohydrates in the source leaves reduce the photosynthetic efficiency through the negative feedback regulation of the photosynthetic mechanism (Pego et al., 2000), and the structure and function of the photosynthetic organs are also damaged, ultimately inhibiting the synthesis of photosynthates (Samborska et al., 2018). Peng et al. (2018) showed that low Mg treatment inhibited *SWEET* gene expression in soybean, thus obstructing the transport of sucrose to the roots. The decrease in root sucrose content further inhibited root growth and N uptake (Peng et al., 2020b). Hermans et al. (2005) suggested that after 11 days of the low-Mg stress, there is a huge reduction in the translocation of sucrose in sugar beet, and the sucrose transport rate could be restored to the Mg-sufficient level with resupplying Mg for 12–24 h transiently (Cakmak et al., 1994; Cakmak and Kirkby, 2008). The effect of Mg application on improving the quality of different crops is also different. Ceylan et al. (2016) showed that Mg application could promote the crude protein content and crude gluten content of wheat. Jezek et al. (2015) found that Mg application could improve the photosynthetic rate of maize and increase the content of soluble sugar and fat in maize. Chapagain and Wiesman (2004) showed that Mg application could increase the soluble solids and vitamin C content of tomatoes and also improve the appearance quality of tomatoes.

Anthocyanins not only determine the color of red-skinned apples and affect fruit appearance quality but also are a substance beneficial to human health. The biosynthesis of anthocyanin in peels is regulated by the content of N and sugar. Wang et al. (2020a) showed that the biosynthesis of anthocyanin was promoted with the reduction in N content in fruits. Kühn et al. (2011) also obtained similar results. In addition, the contents of sucrose, sorbitol, glucose, and fructose in fruits were significantly positively correlated with the content of anthocyanin, so the biosynthesis of anthocyanin was closely related to C and N metabolism (Jiang et al., 2020; Liao et al., 2022).

China is the world’s largest producer of apples, but it has not paid enough attention to the application of Mg. Currently, the reduction in fruit quality is the main problem in China’s apple industry, and the formation of fruit quality is directly related to C–N metabolism, whereas Mg plays a significant role in regulating C–N metabolism. Therefore, the deficiency of Mg may be a potential factor leading to the reduction in apple fruit quality. Therefore, in this study, the effects of Mg on apple C–N metabolism and fruit quality were investigated to provide a scientific basis for improving the fruit quality during apple production.

## Materials and methods

### Experimental materials

This study was conducted in an apple orchard at Laishan district, Yantai City, Shandong Province, China (121°42′55′′E, 37°49′58′′N), during the fruit expansion stage in 2018 and 2019. Thirty ‘Yanfu3’/M26/Malus *hupehensis* Rehd. apple trees were used as experimental material in this study. Trees were planted in the year 2013 in rows spaced 1.5 m apart with 4 m between the rows and were trained as a slender spindle. The density of the experimental site was 111 apple trees per 667 m^2^. The mean temperatures in August, September, and October were 27.3°C, 22.1°C, and 14.6°C in 2018 and 27.1°C, 21.8°C, and 14.3°C in 2019, respectively. The precipitation in August, September, and October was 126.6, 52.2, and 4.1 mm in 2018 and 124.2, 51.3, and 3.5 mm in 2019, respectively. The soil was brown loam, the pH of soil was 5.77, the soil organic matter content was 12.56 g/kg, and available potassium (K), available phosphorus (P), NO_3_-N, and ${\text{NH}}_{4}^{+}-\text{N}$ were 211.23, 59.51, 45.73, and 29.31 mg/kg, respectively.

### Experimental design

Thirty apple trees with the same crop loads (5.5–6.2 fruit per cm^2^ cross-sectional area of fruiting branch), developmental attributes, and number of autumn branches were selected to reduce the individual differences in the sources of photoassimilates in the fruits. In the experiment, there were five treatments, namely, CK, Mg_50_, Mg_100_, Mg_150_, and Mg_200_, which represented 0, 50, 100, 150, and 200 kg/ha of pure Mg application (equal to 0, 250, 500, 750, and 1,000 kg/ha of MgSO_4_), respectively. The date of flowering was 25th April, and the date of fruit maturity stage was 20th October.

To exclude the natural abundance of ^13^C and ^15^N in the apple trees affected by different Mg treatments, each treatment was divided into two groups of three replicates, the first group for isotope labeling and the second for the determination of natural abundance and other indicators. Fertilization treatment was conducted on 1st August (95 days after blooming) in 2018 and 2019. Each tree of group 1 was supplied with 100 g of common urea and 20 g of ^15^N-urea (abundance of 10.28%), each tree of group 2 was supplied with 120 g of common urea (200 kg/ha of pure N), and a circular ditch with a depth and width of 20 cm was dug 40 cm away from the center trunk when fertilizing. MgSO_4_, as the only Mg source, mixed with urea/(^15^N-urea) was evenly watered in the circular ditch after being dissolved in water. All apple trees were subjected to destructive sampling at the 20th October (180 days after blooming, the period of fruit maturity stage).

### ^15^N and ^13^C labeling method

^15^N labeling was performed on 1st August (95 days after blooming). Group 1 of each treatment was supplemented with 20 g of ^15^N-urea and 100 g of common urea, which were mixed and solutioned with MgSO_4_ and then fertilized to the soil. The whole plant was destructively sampled on 20th October (180 days after flowering), and then the indexes correlated with ^15^N were determined.

^13^C isotope labeling was performed on 17th October (177 days after flowering). Fans, a beaker with 8 g of Ba^13^CO_3_ and reduced iron powder, and the labeled whole apple tree were placed into a labeling chamber, which was composed of 0.1-mm-thick Mylar plastic bags and bracket. The light intensity inside was 90% of the natural light intensity. The ^13^C isotope labeling was performed at 8.30 a.m.; the fan was turned on, and the labeling room was sealed. To maintain a suitable temperature (25°C–35°C), an appropriate amount of ice was placed in the labeling room. To maintain a suitable concentration of CO_2_, we injected 1 ml hydrochloric acid into the labeling room by a syringe for every 30 min. The whole plant was destructively after 72 h (180 days after flowering), and then the indexes correlated with ^13^C were determined.

### ^15^N and ^13^C concentration determination

The apple trees were divided into different organs (fruits, roots, perennial branches, trunk, annual branches, and leaves) and the fresh weight weighed; partial samples were then taken with the fresh weight weighed, then washed with clear water and dried at 105 °C for 30 min and 80 °C for 72 h, and then the dry weight was weighed to calculate the water content of each organ; the dry weight of each organ = the fresh weight of each organ × the water content of each organ. Subsequently, the samples were crushed by an electric mill and passed through a 60-mesh sieve (Sha et al., 2020). The content of N and the abundance of ^15^N were determined with a ZHT-03 mass spectrometer (Beijing Analytical Instrument factory). The δ13C values were determined with a DELTA V advantage isotope ratio mass spectrometer (Thermo Fisher, China). Six biological replicates and three technical repetitions were conducted for each treatment.

### Calculation of ^15^N


(1) $$\text{Ndff}(\%)=\frac{{\text{abundance of}}^{15}\text{N in plant}-{\text{natural abundance of}}^{15}\text{N}}{{\text{abundance of}}^{15}\text{N in fertilizer}-{\text{natural abundance of}}^{15}\text{N}}\times 100\%$$

(2)  N15 absorbed by each organ from fertilizer(mg)=Ndff(%)×organ total nitorgen(mg)

(3)  N15 allocation rate(%) N15 absorbed by each organ from fertilizer(mg)total  N15 absorbed by plant from fertilizer(mg)×100%

### Calculation of ^13^C


(4) $$Abundance\;of^{13}C:\;F_{i}\left(\%\right)\;=\frac{({\text{δ}}^{13}\text{C}+1000)\times {\text{R}}_{\text{PBD}}}{({\text{δ}}^{13}\text{C}+1000)\times {\text{R}}_{\text{PBD}}+1000}\times 100\%$$

(5) $$\begin{array}{l} {\text{R}}_{\text{PBD}}\left(\text{standard ratio of carbon isotope}\right)=0.0112372\\ \text{Carbon content of each organ}:{\text{ C}}_{\text{i}}=\text{amount of dry matter}\left(\text{g}\right)\times \text{total carbon content}\left(\%\right) \end{array}$$

(6) $${\text{Content of }}^{13}\text{C of each organ}{:}^{13}{\text{C}}_{i}\left(\text{mg}\right)\;=\frac{{\text{C}}_{\text{i}}\times ({\text{F}}_{i}-{\text{F}}_{nl})}{100}\times 1000$$

F*_nl_*: no ^13^C labeling, natural abundance of ^13^C of each organ


(7)  C13 allocation rate:13C(%)= Ci13 Cnet absorbtion13×100%

### N and Mg concentration determination

The samples were weighed and wet digested in concentrated HNO_3_–H_2_O_2_ at 100°C, 140°C, and 160°C for 1.5 h each until no brown fume appeared, and then further digested at 200°C until the digest became clear. Mg content was analyzed by a spectrometer (ICP-MS systems, Agilent No. 7500 Series). The concentration of N was measured by the Tian et al. (2023) method. Thirty replicates were conducted for each treatment.

### Photosynthetic parameter determination

The net photosynthetic rate (P_n_), transpiration rate (T_r_), stomatal conductance (G_s_), and intercellular CO_2_ concentration (C_i_) were measured by a Li-6400 photosynthetic instrument (LI-COR Inc., USA) from 8:30 to 10:30 a.m. under standardized climatic conditions; the light-saturation point was set to 1,200 μmol (photon)·mÀ 2·sÀ 1, the ambient temperature of the apple leaves was kept constant at 30°C, the CO_2_ concentration was 400 μmol (CO_2_)·molÀ 1, relative humidity was 60%–65%, and air flow was 500 μmol sÀ 1. The chlorophyll content was measured, as described by Wen et al. (2019). Rubisco activity was measured by the method described by Liu et al. (2013).

### Determination of the concentrations of sorbitol, sucrose, fructose, and glucose

Sorbitol, sucrose, fructose, and glucose concentrations were determined, as described by Ma et al. (2019). The sample was weighed for 1 g and treated with 4 ml of 75% ethanol, then heated at 70°C for 12 min in a water bath; the mixed liquor was centrifuged at 5,500g for 8 min, the supernatants were taken out and then treated with 4 ml of 75% ethanol and centrifuged again. The supernatant from the two centrifugations was amalgamated and dried, and the residual was resuspended in 2.5 ml of distilled water and desalinized using an ion-exchange resin column (Selion SBA 2000). The sucrose, fructose, glucose, and sorbitol contents were determined by filtration using liquid chromatography methods (Filip et al., 2016).

### Enzyme activity determination

Nitrate reductase (NR), glutamine synthetase (GS), and glutamate synthase (GOGAT) contents were determined following the method of Hu et al. (2016). Nitrite reductase (NiR) activity was assayed according to the method previously described (Seith et al., 1994).

The activity of C-metabolizing enzymes was measured as follows. For the preparation of enzyme solution, 1 g of the fruit sample was ground into an ice bath in a precooled mortar; 100 mol·l^-1^ of 5 ml Tris–HCl (pH 7.0) buffer, containing 2% glycol, 2 mol·l^-1^ EDTA, 5 mol·l^-1^ MgCl_2_, 2% PVPP, 2% bovine serum protein (BSA), and 5 mmol·L^-1^ DTT, was added for fractional times. 3 ml of the supernatant was put into a dialysis bag after centrifugation at 4°C and 10,000 r·min^-1^ for 20 min. The extraction buffer diluted five times (removing PVPP) was used for dialysis for 15 to 24 h at low temperature (2°C–4°C). The enzyme solution after dialysis was used for determination of various enzyme activities. Sorbitol dehydrogenase (SDH) activity was determined as described by Rufly and Huber (1983). Sorbitol oxidase (SOX) activity was determined as described by Yamaki and Asakura (1991). Sucrose synthase decomposition direction activity (SS-c) was determined, as described by Huber (1983). Sucrose synthase (SS) and sucrose phosphate synthase (SPS) activities were determined as described by Xu et al. (2012). The activities of acid invertase (AI) and neutral invertase (NI) were determined as described by Merlo and Passera (1991).

### The content of ${\text{NO}}_{3}^{-},{\text{NO}}_{2}^{-},{\text{NH}}_{4}^{+}$ , free amino acid, and soluble protein determination

0.1 g of fresh samples was crushed in 1 ml deionized water; the homogenates were then incubated for 30 min in a boiling water bath. After cooling, they were centrifuged at 25°C, 12,000g, for 15 min, and the supernatant was taken to be tested. The ${\text{NO}}_{3}^{-}$ content was measured by nitration of salicylic acid (Cataldo et al., 1975).

0.1 g of fresh samples was homogenized in an extraction solution (1 ml of 100 145 mM HCI and 500 µl of chloroform) and centrifuged at 12,000g at 4 °C for 10 min, and the supernatant was taken to be tested. The ${\text{NH}}_{4}^{+}$ content was determined using the method of Brautigam et al. (2007). The ${\text{NO}}_{2}^{-}$ content in roots and leaves was analyzed as described by Gou et al. (2020).

0.2 g of fresh samples was crushed in 5 ml cold phosphate buffer (50 mM of 150 KH_2_PO_4_, pH 7) and centrifuged at 12,000 g for 15 min. The supernatant was used for analysis. The free amino acid and soluble protein content were measured according to the method of Ruiz and Romero (2002).

### RNA isolation and qRT-PCR analysis

According to the previous research and the purpose of our experiment (Peng et al., 2020a; Wang et al., 2020a; Sha et al., 2020), three *MdSOT*s, three *MdSUT*s, and seven genes related to anthocyanin biosynthesis were selected to determine the relative expression. Total RNA was extracted from around 60 mg of fresh leaves or roots tissues using RNAiso Plus (Takara, Otsu, Shiga, Japan). cDNA synthesis was performed using the ReverTra Ace^®^ qPCR RT Master Mix with gDNA Remover (TOYOBO, Osaka, Japan). Gene relative expression was analyzed by RT-qPCR on a LightCycler 96 (Roche, Basel, Switzerland) using the TranStart Top Green qPCR SuperMix (TransGen Biotech). The actin gene was used as an internal control. Six biological replicates and three technical replicates were determined for each treatment. Expression was normalized by the 2^-ΔΔCt^ method. The qRT-PCR primers are listed in **Table S1**.

### Fruit quality determination

The concentration of soluble sugar in fruit was measured as described by Liu et al. (2018). The sample of apple flesh was placed in a test tube, then 5 ml of distilled water was added and mixed after cutting it into chunks. The supernatant was collected after 30 min of boiling water bath. This step was repeated twice, using distilled water to adjust the volume of the solution to 10 ml. The absorbance of the solution was measured at 630 nm after adding sulfuric acid and anthrone. Six biological replicates and three technical repetitions were conducted for each treatment.

The concentration of anthocyanin was measured as described by Zheng et al. (2021). The sample of fruit peel was ground with liquid nitrogen rapidly and then extracted with 1.5 ml of 1% (v/v) HCl methanol at 3°C for 30 h in darkness. The concentration of anthocyanin was measured by a multifunctional microplate reader (BioTek, USA). Six biological replicates and three technical repetitions were conducted for each treatment.

The concentration of titratable acid was measured by NaOH titration (Nie et al., 2012), and the fruit hardness was measured by a HP-230 hardness tester. The diameter of fruit was measured with a vernier caliper. Single fruit weight was measured by 1% precision electronic balance, and the average of 30 measured values was taken.

### Statistical analysis

The figures were prepared using Origin 2019b (OriginLab Corporation, USA). The contents of N and Mg in fruits and leaves were determined for 30 biological replicates and three technical repetitions; other indexes were determined for six biological replicates and three technical repetitions. Data were analyzed with SPSS 17.0 (Statistics software, version 20.0, IBM, USA) using one-way analysis of variance (ANOVA). The significant differences were considered at a probability level of P ≤ 0.05.

## Results

### Effects of Mg application on fruit quality

As presented in **Table 1**, the application of Mg significantly improved the fruit yield and quality. Fruit yield increased with the increase in Mg application, compared with CK treatment; the yield in Mg_150_ treatment was increased by 6.86% and 7.29% in 2018 and 2019, respectively. In addition, with the increase in the application of Mg, the single fruit mass, transverse diameter, longitudinal diameter, soluble sugar, and sugar–acid ratio were all increased and reached the highest value at Mg_150_ treatment, which increased by 7.77%, 4.35%, 5.06%, 19.07%, and 19.05% (2018) and 7.29%, 6.93%, 4.48%, 16.89%, and 16.89% (2019) higher than those under CK treatment, respectively. However, there was no significant difference in fruit firmness and the content of titratable acid among different treatments.

**Table 1:** Effects of Mg application on fruit quality at the fruit maturity stage in 2018 and 2019.

Year	Treatment (kg/hm^2^)	Yield (kg/667 m^2^)	Single fruit mass (g)	Transverse diameter (mm)	Longitudinal diameter (mm)	Fruits firmness (kg·cm^-2^)	Soluble sugar (%)	Titratable acid (%)	Sugar–acid ratio
2018	CK	2622.2c	234.23c	80.6b	71.2b	8.36a	12.53c	0.46a	27.24c
	Mg_50_	2714.7b	244.57b	81.2b	71.8b	8.21a	13.95b	0.48a	29.06b
	Mg_100_	2798.6a	252.13a	81.6b	71.3b	8.07a	14.07b	0.48a	29.31b
	Mg_150_	2802.0a	252.43a	86.1a	74.8a	8.08a	14.92a	0.46a	32.43a
	Mg_200_	2795.6a	251.86a	85.7a	74.3a	7.92a	14.83a	0.46a	32.24a
2019	CK	2646.5c	236.42c	80.8b	71.4b	8.13a	12.61c	0.46a	27.41c
	Mg_50_	2743.0b	247.12b	81.6b	71.9b	8.27a	13.77b	0.47a	29.3b
	Mg_100_	2835.3a	255.43a	81.9b	71.1b	7.82a	14.68a	0.48a	30.58a
	Mg_150_	2839.5a	255.81a	86.4a	74.6a	8.11a	14.74a	0.46a	32.04a
	Mg_200_	2832.4a	255.17a	86.1a	74.2a	7.96a	14.73a	0.46a	32.02a

Significant differences were detected by different letters at P ≤ 0.05.

### Effects of Mg application on anthocyanin content and anthocyanin biosynthesis-related gene expression in the peel

**Figure 1:**
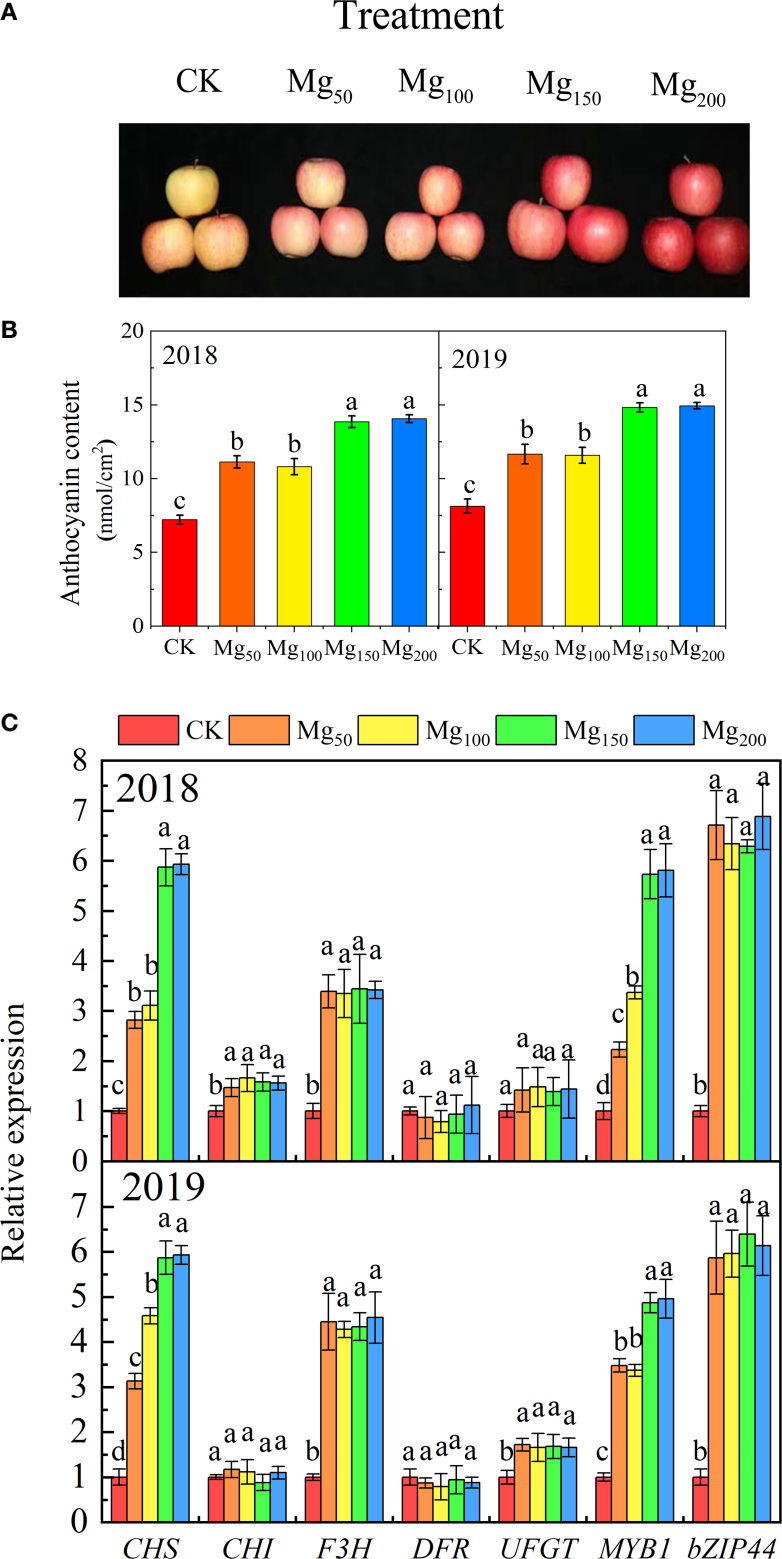
Effects of Mg application on fruit peel coloring. A representative photograph **(A)** shows the color of the fruit peel under different Mg treatments. Anthocyanin concentration **(B)** and anthocyanin biosynthesis-related gene expression **(C)** of fruit peel were measured in each treatment. The error bars indicate SD of six replications. Significant differences were detected by different letters at P ≤ 0.05.

As shown in **Figure 1**, we found that the application of Mg significantly promoted fruit coloring (**Figure 1A**). To further verify the discovery, we measured the expression of genes (*MdCHS*, *MdCHI*, *MdF3H*, *MdDFR*, and *MdUFGT*) and transcription factors (*MdMYB1* and *MdbZIP44*) associated with anthocyanin synthesis in peel. The results showed that the application of Mg significantly upregulated the expression of *MdCHS*, *MdF3H*, *MdMYB1*, and *MdbZIP44* (**Figure 1C**). The content of anthocyanin also increased with the increased application of Mg (**Figure 1B**) and reached the highest values at Mg_150_ and Mg_200_ treatments, which were 91.97% and 94.88% (2018) and 82.41% and 83.76% (2019) higher than that at CK treatment, respectively.

### Effects of Mg application on the content of N and Mg in leaves and fruits

**Figure 2:**
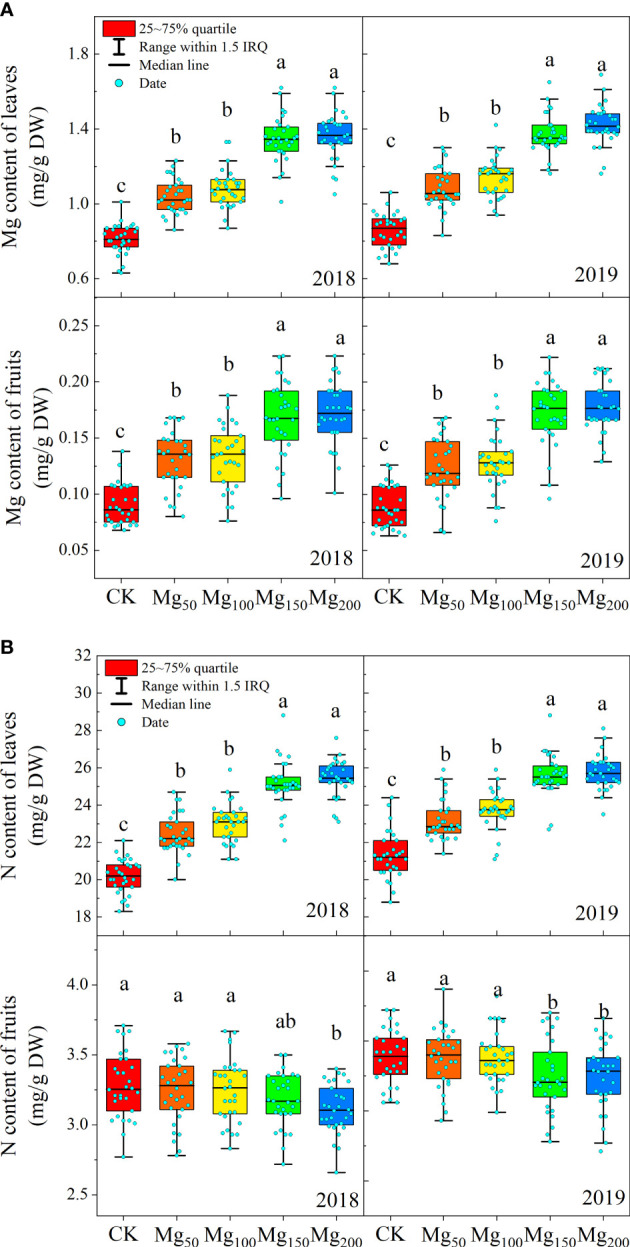
Effects of Mg application on the content of Mg **(A)** and N **(B)** in the leaves and fruits in 2018 and 2019. The bars indicate the range within 1.5 IRQ of 30 replications, the box represents 25 to 75% quartile, and the line in the box indicates the median line. Significant differences were detected by different letters at P ≤ 0.05.

Different Mg treatments significantly affected the contents of N and Mg in leaves and fruits. As shown in **Figure 2**, with the increase in Mg application, the content of Mg both in leaves and fruits showed a significant increase. The content of N was also increased significantly in leaves and reached the maximum value at Mg_150_ and Mg_200_ treatments, and there was no significant difference between Mg_150_ and Mg_200_ treatments. On the contrary, the N content of fruits decreased with the increase in Mg application.

### Effects of Mg application on photosynthesis and C metabolism

#### Effects of Mg application on photosynthetic parameter

**Figure 3:**
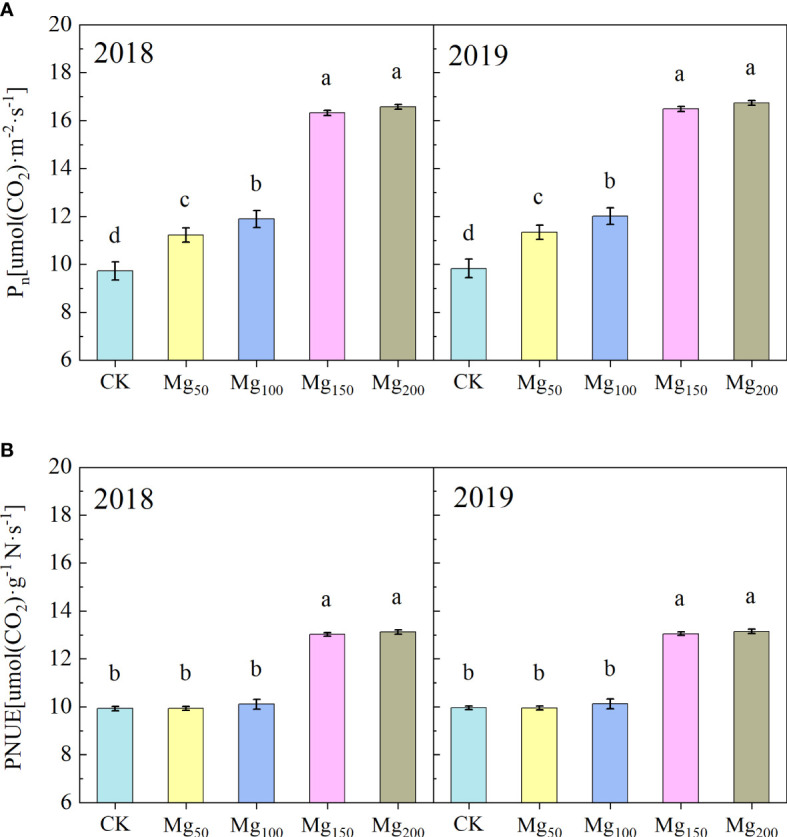
Effects of Mg application on the net photosynthetic rate (P_n_) **(A)** and photosynthetic nitrogen use efficiency (PNUE) **(B)** in the leaves in 2018 and 2019. The bars represent mean ± SE, n = 6. Different letters on vertical bars indicate significant differences (P< 0.05).

As presented in **Table 2**, 2 years of repeated experiments showed that different Mg treatments significantly affected the photosynthetic performance of leaves. The treatments of Mg_150_ and Mg_200_ enhanced the chlorophyll content and Rubisco activity to varying degrees, compared with CK, with increases of 79.77%–95.49% and 64.86%–69.44% in both years, respectively. In addition, the application of Mg also significantly increased the transpiration rate (T_r_), stomatal conductance (G_s_), and intercellular CO_2_ concentration (C_i_). In addition, P_n_ and PNUE of leaves were also significantly changed by Mg (**Figure 3**). The values of P_n_ and PNUE under CK treatment were at the lowest level; compared with CK treatment, P_n_ significantly increased under Mg_50_ and Mg_100_ treatment, whereas PNUE had no significant difference. With further increase in Mg application (150–200 kg/ha), PNUE had significantly increased.

**Table 2:** Effects of Mg application on chlorophyll content, rubisco activity, transpiration rate (T_r_), stomatal conductance (G_s_), and intercellular CO_2_ concentration (C_i_) in 2018 and 2019.

Year	Treatment (kg/hm^2^)	Chlorophyll content (mg·g^-1^ FW)	Rubisco activity (μmol·g^-1^·min^-1^ FW)	T_r_ (mmol·m^-2^·s^-1^)	G_s_ (mol·m^-2^·s^-1^)	C_i_ (μmol·mol^-1^)
2018	CK	3.41 ± 0.18d	5.89 ± 0.41d	8.31 ± 0.44b	0.28 ± 0.01c	263.3 ± 11.26c
	Mg_50_	4.31 ± 0.11c	7.12 ± 0.33c	9.01 ± 0.56a	0.34 ± 0.01b	279.4 ± 16.12b
	Mg_100_	5.28 ± 0.07b	8.34 ± 0.36b	9.07 ± 0.52a	0.33 ± 0.01b	283.6 ± 7.36b
	Mg_150_	6.13 ± 0.24a	9.71 ± 0.18a	9.21 ± 0.43a	0.41 ± 0.01a	298.2 ± 14.11a
	Mg_200_	6.04 ± 0.22a	9.58 ± 0.41a	9.13 ± 0.51a	0.42 ± 0.02a	295.5 ± 11.66a
2019	CK	3.55 ± 0.26c	5.89 ± 0.41d	8.31 ± 0.74c	0.28 ± 0.014d	265.2 ± 16.33d
	Mg_50_	5.04 ± 0.21b	7.12 ± 0.33c	9.02 ± 0.76b	0.35 ± 0.007c	272.4 ± 15.88c
	Mg_100_	5.66 ± 0.48b	8.34 ± 0.36b	9.11 ± 0.83b	0.34 ± 0.009c	282.5 ± 17.51bc
	Mg_150_	6.94 ± 0.31a	9.98 ± 0.41a	9.34 ± 0.81a	0.40 ± 0.015a	301.2 ± 17.85a
	Mg_200_	6.73 ± 0.32a	9.71 ± 0.38a	9.25 ± 0.73ab	0.38 ± 0.012ab	287.5 ± 15.03b

Significant differences were detected by different letters at P ≤ 0.05.

#### Effects of Mg application on δ^13^C and ^13^C distribution rates

**Figure 4:**
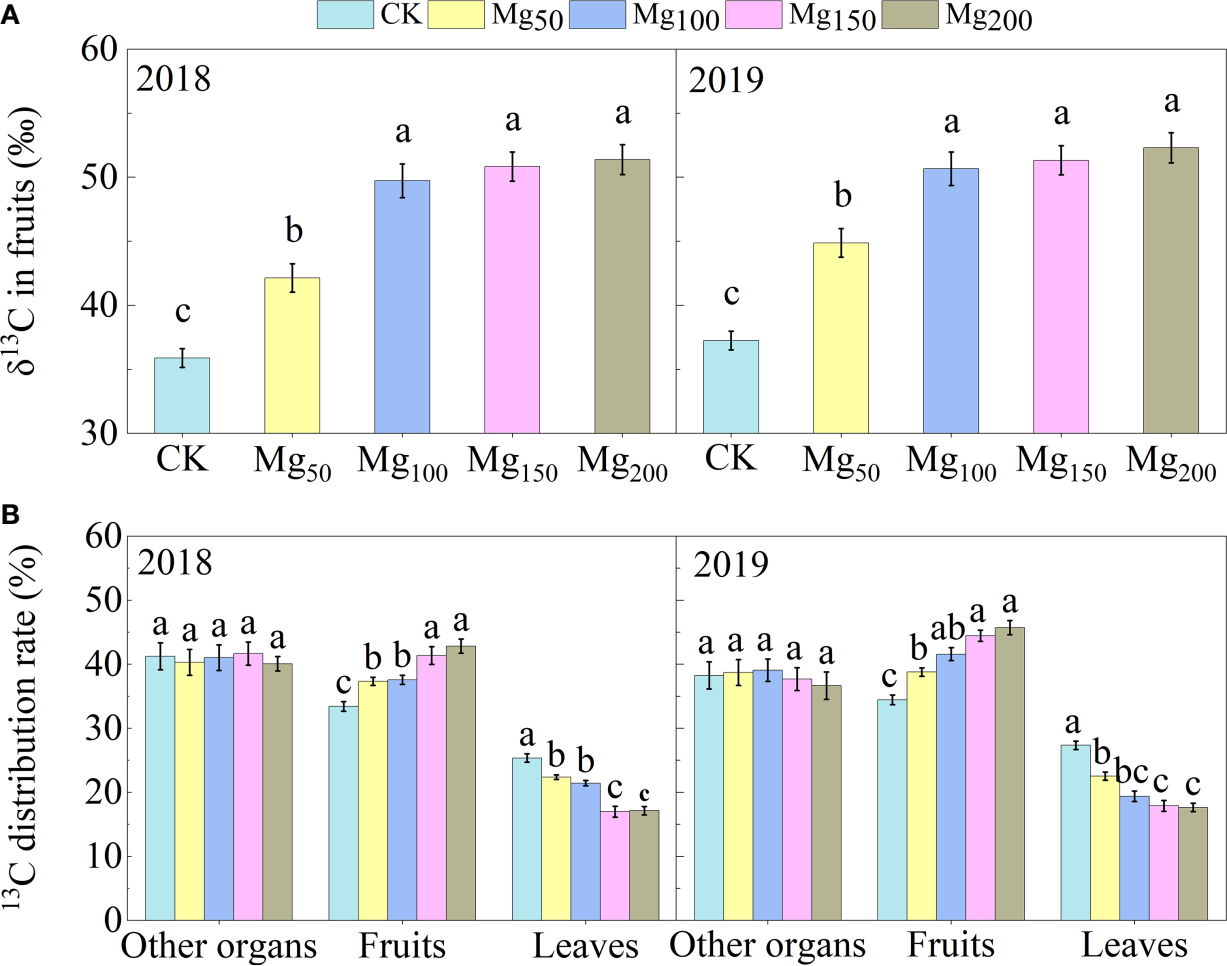
δ^13^C value in fruit **(A)** and ^13^C distribution rate in different organs **(B)** of the ‘Red Fuji’ apple tree were measured under Mg_0_, Mg_50_, Mg_100_, Mg_150_, and Mg_200_ treatments in 2018 and 2019. The bars represent mean ± SE, n = 6. Different letters on vertical bars indicate significant differences (P< 0.05).

^13^C isotope tracer technology showed that different Mg applications changed the ^13^C accumulation of fruits (**Figure 4**). Under CK treatment, the δ^13^C values of fruits were at the lowest level, which were 35.88‰ (2018) and 37.24‰ (2019). With the application of Mg reaching 150–200 kg/ha, δ^13^C of fruits significantly increased by 43.2% (2018) and 40.4% (2019) compared with the CK treatment, respectively. In addition, the allocation of ^13^C in different organs was analyzed. We found that Mg application significantly increased the distribution of ^13^C in fruits and decreased in leaves but had no significant effect in other organs (roots, trunk, perennial branches, and annual branches), which showed that Mg application could promote the distribution of ^13^C from leaves to fruits.

#### Effects of Mg application on enzyme activities of C metabolism in leaves and fruits

As shown in **Table 3**, with the increase in Mg addition, the activities of sucrose synthase (SS), sucrose phosphate synthase (SPS), and sorbitol 6-phosphate dehydrogenase (S6PDH) in leaves were significantly promoted and Mg could also significantly improve the activities of sorbitol dehydrogenase (NAD^+^-SDH), sucrose synthase cleavage direction (SS-c), neutral invertase (NI), and acid invertase (AI) in fruits. However, the activities of sorbitol oxidase (SOX) in leaves had no change with Mg application, indicating that Mg could promote the synthesis of sucrose and sorbitol in leaves and the decomposition of sorbitol and sucrose in fruits.

**Table 3:** Effects of Mg application on enzyme activities of C metabolism in fruit (NAD^+^-SDH, SOX, SS-c, NI and AI) and leaf (SS, SPS, S6PDH).

Year	Treatment (kg/hm^2^)	Enzyme activities in fruit [µmol·g^-1^·h^-1^]					Enzyme activities in leaf [μg·min^-1^·g^-1^]		
		NAD^+^-SDH	SOX	SS-c	NI	AI	SS	SPS	S6PDH
2018	CK	0.95c	3.33a	8.13c	3.57d	11.12b	15.42b	325.6d	322.9c
	Mg_50_	1.62b	3.57a	8.94b	4.27c	12.48a	29.11a	457.3c	437.6b
	Mg_100_	1.67b	3.58a	9.02b	4.31c	12.44a	29.85a	475.2c	451.3b
	Mg_150_	2.23a	3.42a	9.74a	4.73b	12.47a	30.27a	528.1b	688.2a
	Mg_200_	2.26a	3.57a	9.85a	4.76a	12.54a	31.32a	586.7a	698.7a
2019	CK	0.88c	3.28a	8.06c	3.48c	11.03c	15.42c	335.8c	335.7d
	Mg_50_	1.52b	3.15a	8.84b	4.19b	11.66b	22.92b	465.5b	448.2c
	Mg_100_	1.57b	3.32a	8.75b	4.25b	12.27a	29.66a	468.3b	574.5b
	Mg_150_	2.05a	3.46a	9.66a	4.58a	12.32a	30.16a	578.1a	690.1a
	Mg_200_	2.12a	3.63a	9.78a	4.69a	12.34a	31.24a	593.4a	688.3a

Significant differences were detected by different letters at P ≤ 0.05.

#### Effects of Mg application on the content of soluble sugar in leaves and fruits

**Figure 5:**
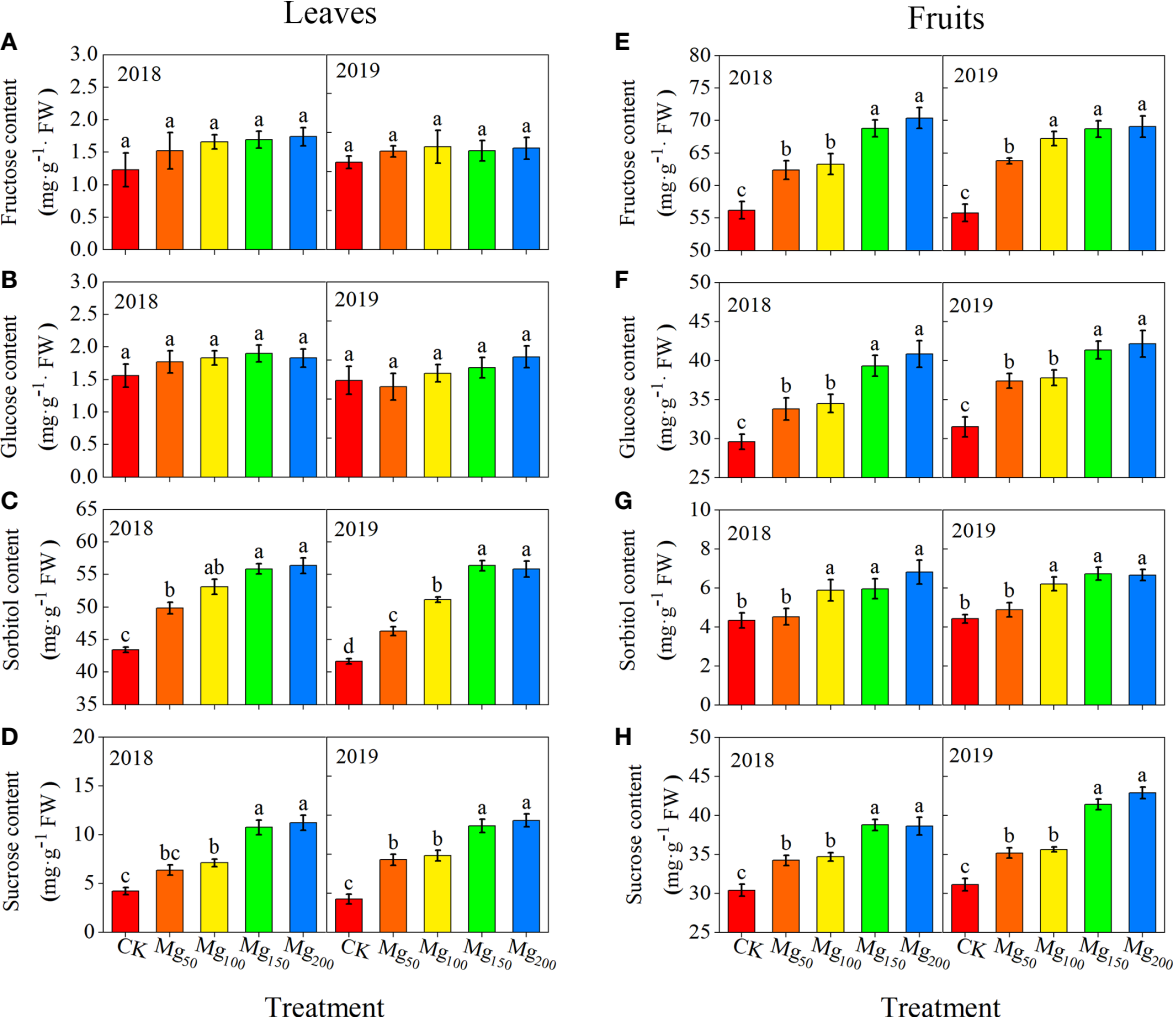
Effects of Mg application on the content of fructose **(A, E)**, glucose **(B, F)**, sorbitol **(C, G)**, and sucrose **(D, H)** in leaf **(A-D)** and fruit **(E-H)** in 2018 and 2019. The error bars indicate SD of six replications. Significant differences were detected by different letters at P ≤ 0.05.

The concentrations of sucrose, sorbitol, fructose, and glucose showed to be different in fruits and leaves (**Figure 5**). Under CK treatment, the content of sorbitol was the highest in leaves, followed by sucrose, whereas fructose and glucose contents were at the lowest level. On the contrary, the fructose content in fruits was the highest, followed by glucose and sucrose, and finally sorbitol. Different Mg treatments changed the sugar content in leaves and fruits; with the increase in Mg application, the contents of sorbitol and sucrose in leaves were significantly enhanced whereas there was no change in the contents of fructose and glucose. Compared with the CK treatment, the concentration of sorbitol and sucrose increased at 28.67%–31.53% and 155.34%–238.77% under Mg_150_ and Mg_200_ treatments for both years, respectively. Moreover, the application of Mg had no change in sorbitol concentration, and the treatments of Mg_150_ and Mg_200_ enhanced the sucrose, fructose, and glucose concentrations to varying degrees, compared with CK, with increases of 12.89%–37.87%, 22.42%–25.27%, and 31%–33.81% in both years, respectively.

#### Effects of Mg application on the gene expression of sugar transporter

**Figure 6:**
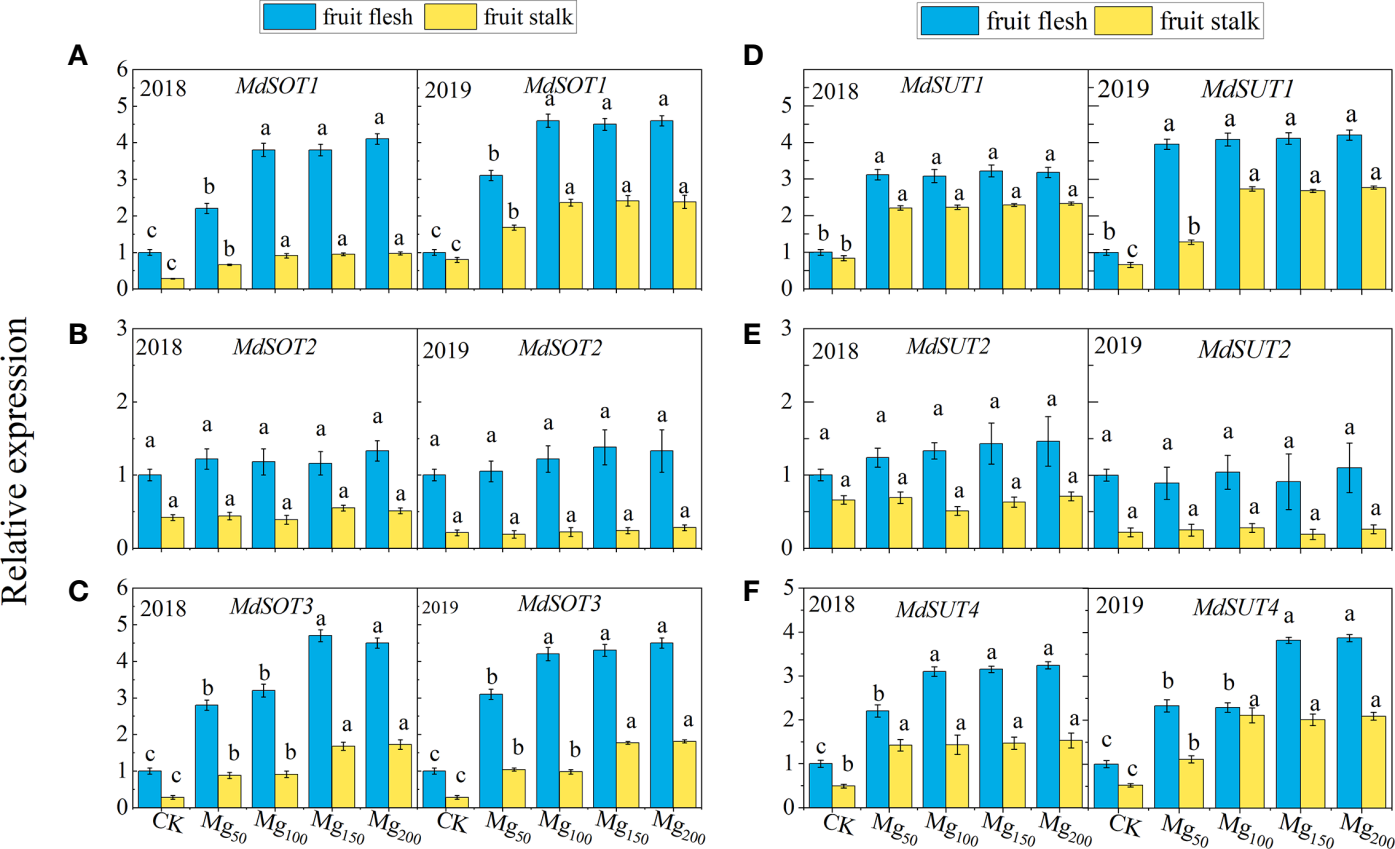
Effects of Mg application on the relative expression of *MdSOT1* **(A)**, *MdSOT2* **(B)**, *MdSOT3* **(C)**, *MdSUT1* **(D)**, *MdSUT2* **(E)**, and *MdSUT4* **(F)** in fruit stalk and fruit flesh in 2018 and 2019. The bars represent mean ± SE, n = 6. Different letters on vertical bars indicate significant differences (P< 0.05).

To further investigate the effect of Mg application on sorbitol and sucrose transport to fruits, we determined the expression of genes related to sugar transport (**Figure 6**), and the results showed that *MdSOT1/3* and *MdSUT1/4* were significantly upregulated by Mg under Mg_150_ and Mg_200_ treatments. The results indicated that sufficient Mg promoted sorbitol transport to fruits by inducing *MgSOT1/3* expression and promoted sucrose transport to fruits by upregulating *MdSUT1/4*. These results may explain the distribution of ^13^C between leaves and fruits to some extent.

### Effects of Mg application on N metabolism

#### Effects of Mg application on ^15^N utilization and distribution

**Figure 7:**
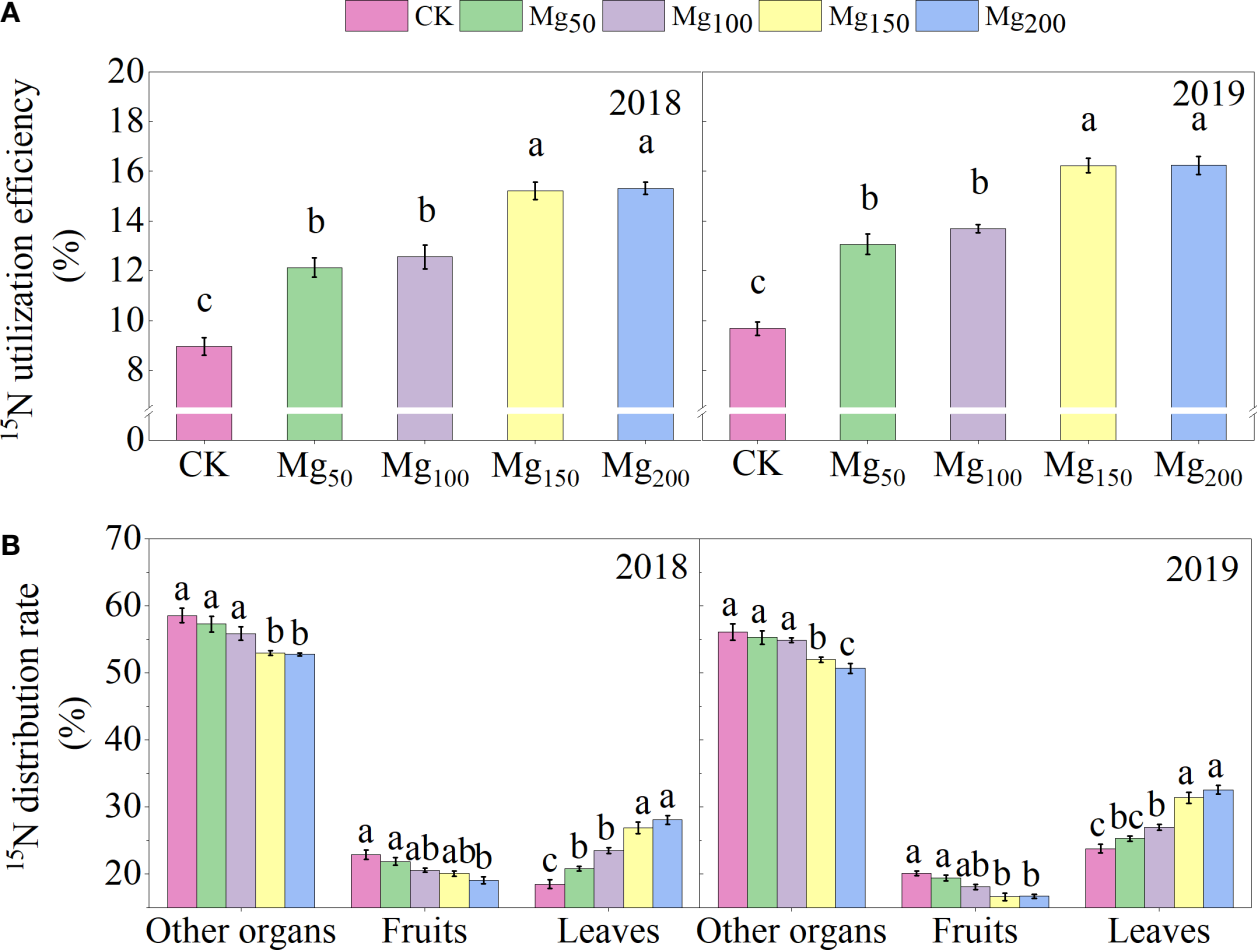
Effects of Mg application on ^15^NUE **(A)** and ^15^N distribution rate **(B)** in different organs of the ‘Red Fuji’ apple tree in 2018 and 2019. The error bars indicate SD of six replications. Significant differences were detected by different letters at P ≤ 0.05.

As shown in **Figure 7**, the ^15^N isotope tracer technique showed that the application of Mg could significantly increase the ^15^N utilization rate of the whole apple tree. Compared with CK treatment, the ^15^N utilization rate under Mg_150_ and Mg_200_ treatments increased by 69.87%–70.99% (2018) and 67.67%–69.01% (2019), respectively. The results showed that the application of Mg increased the absorption of N by apple trees. However, the ^15^N allocation rate showed to be different in organs. The ^15^N allocation rate of leaves increased with the increase in Mg application. On the contrary, the ^15^N allocation rate of fruits and other organs showed a downward trend, which indicated that Mg was conducive to the transfer of ^15^N from other organs to leaves.

#### Effects of Mg application on enzyme activities of N metabolism

**Figure 8:**
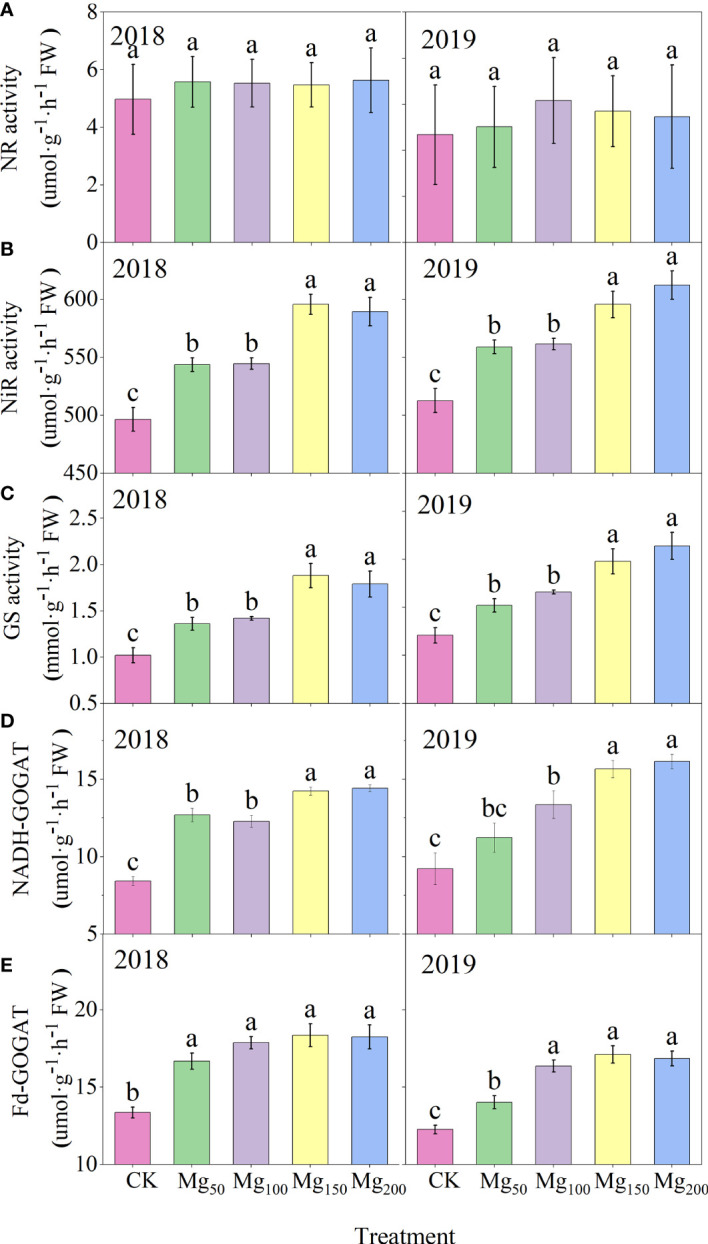
Effects of Mg application on the NR **(A)**, NiR **(B)**, GS **(C)**, NADH-GOGAT **(D)**, and Fd-GOGAT **(E)** activities in leaves in 2018 and 2019. The error bars indicate SD of six replications. Significant differences were detected by different letters at P ≤ 0.05.

Nitrate absorbed by plant roots is reduced into ammonium by nitrate reductase (NR), which is partly carried out in the roots and partly in the leaves. Apples are perennial fruit trees, and nitrate reduction is mostly carried out before being transferred to the leaves, so the NR activity in the leaves is extremely low. As shown in **Figure 8**, different Mg treatments significantly affected the enzyme activities of N metabolism. The enzyme activities of each treatment showed the lowest level with 0 Mg application and increased to the highest level under the application of 150–200 kg/ha Mg, and there was no significant difference between the two treatments. Different enzymes were affected to different degrees, among which glutamine synthase (GS) was the most promoted by 150–200 kg/ha Mg application, and the highest value was 1.84 times (2018) and 1.77 times (2019) of the CK treatment.

#### Effects of Mg application on the content of intermediate products in N metabolism

**Figure 9:**
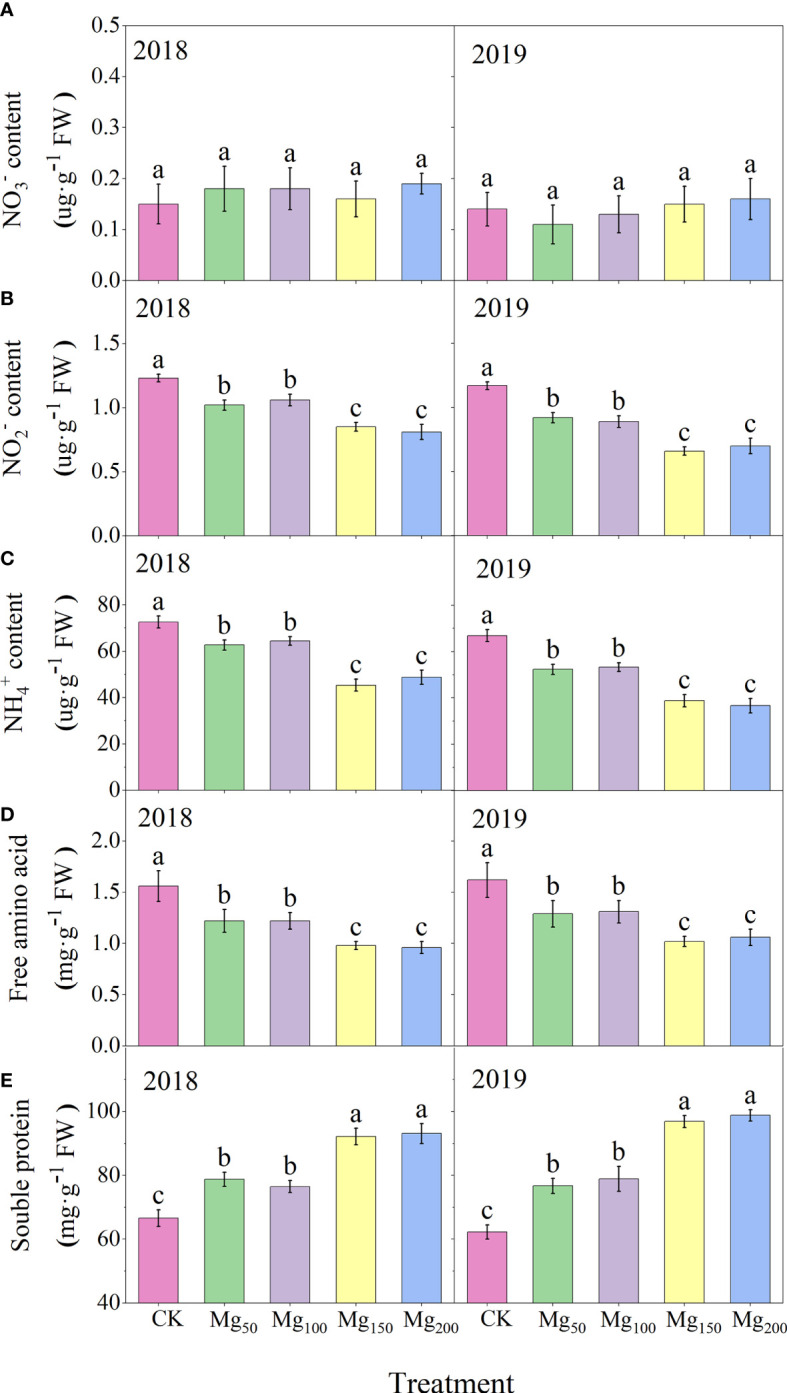
Effects of Mg application on the content of ${\text{NO}}_{3}^{-}$ **(A)**, ${\text{NO}}_{2}^{-}$ **(B)**, ${\text{NH}}_{4}^{+}$ **(C)**, free amino acid **(D)**, and soluble protein **(E)**, in leaves in 2018 and 2019. The error bars indicate SD of six replications. Significant differences were detected by different letters at P ≤ 0.05.

As presented in **Figure 9**, Mg supply had no significant effect on ${\text{NO}}_{3}^{-}$ content in leaves, but it greatly reduced the contents of ${\text{NO}}_{2}^{-}$ and ${\text{NH}}_{4}^{+}$ , due to the fact that Mg improved the activities of NiR and GS and promoted the further transformation of ${\text{NO}}_{2}^{-}$ and ${\text{NH}}_{4}^{+}$ . Glutamine is an important form of N storage in plants; under Mg-deficiency treatment, the ${\text{NH}}_{4}^{+}$ concentration in leaves was higher and the GS activity was improved with the increase in Mg, which promoted the synthesis of ${\text{NH}}_{4}^{+}$ into glutamine in large quantities and effectively prevented the accumulation of ${\text{NH}}_{4}^{+}$ poisoning. In addition, under CK treatment, the content of free amino acid in leaves was the highest, whereas the content of soluble protein was the lowest. The addition of Mg could significantly reduce the content of free amino acid and increase the content of soluble protein.

## Discussion

### The appropriate application of Mg promoted N metabolism

N is an essential nutrient for the growth and development of apple trees. It has an irreplaceable effect on the organ construction, material metabolism, physiological and biochemical processes, and formation of fruit yield and quality (Liu et al., 2022b). The fruit expansion stage is the period when the fruit has the strongest nutrient demand. Insufficient N supply reduces the precursor substances of C metabolism, which is not conducive to tree development and fruit quality. Previous studies have shown that Mg addition can improve the N absorption in tea plants (Jayaganesh and Venkatesan, 2010), rice (Ding et al., 2006), and maize (Szulc et al., 2008). Our research on apples has achieved similar results that appropriate application of Mg significantly increased the Mg and N content of leaves (**Figure 2**), and the ^15^N utilization rate also increased with the increase in Mg application (**Figure 7**), indicating that the application of Mg could increase the absorption of N and improve the NUE in apple trees.

Nitrate reductase (NR), nitrite reductase, (NiR) glutamine synthetase (GS), and glutamate synthase (GOGAT) are key enzymes in the process of N assimilation, and their activity can regulate the key steps of N assimilation. The ${\text{NO}}_{3}^{-}$ absorbed by plants first reduced to ${\text{NO}}_{2}^{-}$ under the action of NR, and this process is carried out in roots or leaves. In our study, both the NR activity and ${\text{NO}}_{3}^{-}$ content of leaves were at a low level (**Figures 8A**, **9A**), and the difference between treatments was not significant, indicating that most ${\text{NO}}_{3}^{-}$ had been reduced to ${\text{NO}}_{2}^{-}$ before being transported to the leaves. Under the action of NiR and GS, ${\text{NO}}_{2}^{-}$ synthesizes ${\text{NH}}_{4}^{+}$ and glutamine, which is then decomposed into glutamate by GOGAT and finally metabolized into other amino acids and N-containing compounds. We found that with the increase in Mg application, the activities of NiR, GS, and GOGAT in leaves increased significantly (**Figures 8B–E**), whereas the contents of N-metabolizing intermediates such as ${\text{NO}}_{2}^{-}$ and ${\text{NH}}_{4}^{+}$ decreased significantly (**Figures 9B–E**), indicating that Mg improved the activity of N-metabolizing enzymes to promote N metabolism and continued to consume N-metabolizing intermediates to promote the conversion of N to amino acids and N compounds. Previous studies also found that Mg can improve the activity of N metabolism enzymes in black tea (Jayaganesh and Venkatesan, 2010) and rice (Ding et al., 2006), which is consistent with our study. Marschner (2012) showed that when the supply of Mg was interrupted, protein synthesis stopped immediately and the process resumed quickly after the resumption of Mg supply, indicating that Mg had a significant effect on protein synthesis. We also found that under adequate Mg supply (Mg_150_–Mg_200_), free amino acid content decreased and soluble protein content increased in leaves (**Figures 9D, E**), indicating that Mg promoted the conversion of free amino acid into soluble protein. Soluble protein is an important component of protoplasts, most of which are key enzymes involved in physiological metabolism. Therefore, the application of Mg promoted N metabolism and increased the synthesis of soluble protein in leaves, which not only promoted the activity of various physiological and biochemical reactions in leaves but also enhanced the demand for N in leaves, thus further enhancing the requisition and allocation of N in leaves. Further analysis showed that the ^15^N allocation rate of leaves increased with the increase in Mg application (**Figure 7B**), whereas the ^15^N allocation rate of perennial organs and fruits decreased, indicating that Mg application effectively promoted the transfer of N from other organs to leaves. In general, the fruit expansion stage is the period of maximum efficiency of N accumulation by fruits; however, it is also the period of the fastest accumulation of fruit biomass. The appropriate application of Mg increased the competition for N in leaves, so the newly absorbed ^15^N allocated to fruits was relatively reduced, which caused N allocated to fruits to not match the rapid growth of fruits; therefore, the ^15^N distribution in fruits (**Figure 4B**) and the N content of fruits (**Figure 2B**) all showed a significant decrease. More N distribution to fruits can decrease the enzyme activities of sugar metabolizing in fruits and decrease fruit quality (Kühn et al., 2011; Sha et al., 2020; Wang et al., 2020a). Therefore, in order to decrease the accumulation of N in fruits, scholars applied nitrification inhibitors 3,4-dimethylpyrazole phosphate (Wang et al., 2020a), abscisic acid (Wang et al., 2020b), and paclobutrazol (Sha et al., 2021) to limit N transport to fruits. We provided a new idea: Mg significantly decreased ^15^N allocated to fruits, which was beneficial to fruit quality to a certain extent. Meanwhile, the allocation of N in various organs also affects the N absorption and assimilation. Mg enhanced the allocation of N to leaves, which is conducive to various physiological and biochemical reactions. The less N allocation in other organs may also further promote the absorption of N through signal stimulation (Chen et al., 2020).

### Mg promoted C metabolism and the transport of photosynthates from leaves to fruits

Photosynthesis realizes the assimilation and distribution of C in plants by using CO_2_ as raw material. In horticultural plants, the accumulation and distribution of photosynthates in different organs affect the fruit quality directly. Compared with Mg deficiency, Mg_150_ and Mg_200_ treatments significantly improved the chlorophyll content, the activity of Rubisco enzymes, P_n_, and PNUE of leaves (**Table 2**; **Figures 3**), thus accelerating the synthesis and accumulation of photosynthates. In addition, Mg_150_ and Mg_200_ treatments significantly increased the enzyme activities of SS, SPS, and S6PDH in leaves (**Table 3**) and the contents of sucrose and sorbitol in leaves also increased by sufficient Mg addition (**Figures 5C, D**), which also meant that Mg promoted the synthesis of sorbitol and sucrose in leaves. Relevant conclusions have been found in soybean (Peng et al., 2018), sugar beet (Poglodzinski et al., 2021), watermelon (Huang et al., 2016), and other crops; these studies are consistent with our results.

The synthesis and transport of photosynthates from leaves to fruits is a very complicated process. CO_2_ is assimilated into propanose phosphate in chloroplasts and then transported into the cytoplasm by related transporters and enzymes; after synthesis of sorbitol and sucrose in the cytoplasm, they are transported over long distances and unloaded into the fruits (Desnoues et al., 2018). Therefore, the effective transfer and distribution of photosynthates to fruits is a key factor affecting fruit growth and development (Katz et al., 2007; Álvaro et al., 2008). Previous studies have shown that Mg facilitates the transport of photosynthates in beet (Hermans et al., 2005), beans (Cakmak and Kirkby, 2008), tea plants (Ruan et al., 2012), and wax gourd (Zhang et al., 2020a). Sucrose is the main photosynthate of most plants, but there is some difference in apple plants; compared with sucrose, sorbitol is the main photosynthate of apples, which accounts for 80% and sucrose only 20% (Chong and Taper, 1971). Although different crops are associated with different photosynthates, Mg has a certain similarity in regulating their transport. The results of ^13^C isotope tracer technology showed that Mg significantly increased the δ^13^C value and ^13^C allocation rate of fruits (**Figure 4**), whereas the ^13^C allocation of leaves decreased, indicating that Mg application promoted the transport of ^13^C photosynthates from leaves to fruits. Subsequently, we determined the expression of key genes involved in sorbitol and sucrose loading and unloading in fruit stalk and fruit flesh. The results showed that adequate application of Mg upregulated the expression of *MdSOT1/3* and *MdSUT1/4* and promoted the loading and unloading of sucrose and sorbitol in phloem (**Figure 6**) and thus promoted the transport efficiency of sugar. After sucrose and sorbitol are transported to the fruits, they are rapidly decomposed into fructose and glucose under the action of related enzymes. We found that appropriate application of Mg could significantly improve the activities of sucrose decomposition enzymes (SS-c, AI, NI) and sorbitol decomposition enzyme (NAD^+^-SDH) in fruits (**Table 3**), indicating that Mg promotes the decomposition of sucrose and sorbitol in the fruits. Therefore, the content of fructose and glucose in fruits also significantly increased in fruits (**Figures 5E, F**). These results are consistent with those of ^13^C isotopic labeling. The application of Mg had not changed SOX activity, but the decomposition of sorbitol had no effect on fruits. It was due to the fact that more than 80% of SOX existed in bound form and its activity was only one-fifth of NAD^+^-SDH, so it had little effect on sorbitol metabolism (Yamaki and Asakura, 1991).

There are two ways of loading and unloading sugar in phloem, namely, the symplastic pathway and the apoplastic pathway (Rennie and Turgeon, 2009). The symplastic pathway refers to the concentration gradient at source and sink organ, and the sugar enters the sink organ through the intercellular desmodesmata between the sieve tube and the companion cell. This process does not involve transmembrane transport and is not limited by energy and transporters (Tegeder and Hammes, 2018). The apoplastic pathway refers to the transmembrane transport of assimilates by sugar transporters, which consumed ATP. Our study suggests that the application of Mg may enhance both pathways and facilitate the transport of sugars from source to sink. 1) The application of Mg significantly promoted the synthesis of sorbitol and sucrose in leaves, increased the content of sugar, and thus increased the source strength (**Figures 5C, D**). Meanwhile, Mg significantly promoted the decomposition of sucrose and sorbitol into glucose and fructose in the sink organ (**Table 3**; **Figure 5**), which not only reduced the osmotic potential in the vacuoles but also increased the concentration gradient of sorbitol at both source and sink organ, and sorbitol ceaselessly entered the sink organ through the cytoplasmic desmata (Eom et al., 2012). Ultimately, the transport efficiency of sorbitol in the symplastic pathway was improved. 2) Different from sorbitol, we found that the sucrose content was higher in the fruits than in the leaves. Previous studies found that this difference was more obvious in the fruit expansion stage (Yang et al., 2019; Sha et al., 2020). Therefore, sucrose transport from leaves to fruits is an inverse concentration gradient process, indicating that sucrose transport depends on energy and sucrose transporters. Mg significantly induced the expression of sugar transporters *MdSOT1/3* and *MdSUT1/4* in fruit stalk and flesh (**Figure 6**), thus promoting the transport of sorbitol and sucrose in the phloem extracellular pathway. Previous studies have shown that low Mg inhibits the transport of carbohydrates from source to sink by downregulating the expression of *BvSUT1* and *SWEET* genes (Hermans et al., 2005; Peng et al., 2018); this is consistent with our results. In addition, the substrate of ATPase is the Mg–ATP complex; the supply of Mg effectively promotes ATP synthesis and provides energy for the loading and unloading of sugars in the apoplastic pathway (Shaul, 2002). Therefore, sufficient Mg promotes the transport of sorbitol and sucrose in the symplastic pathway and also facilitates the transport of sorbitol in the apoplastic pathway.

### Mg improved the anthocyanin biosynthesis and fruit quality

An appropriate application of Mg can increase the yield of corn (Jezek et al., 2015), citrus (Tang et al., 2012b), and potato (Orlovius and Mchoul, 2015). Consistent with previous studies, Mg significantly improved the single fruit weight and transverse and longitudinal diameters of apples (**Table 1**), and the apple fruit yield was also increased with the increase in application of Mg; the highest yield was 2,802 kg/667 m^2^ and 2839.5 kg/667 m^2^ in 2018 and 2019, which were increased by 6.86% and 7.29% than CK treatment, respectively. In addition to fruit yield, Mg also significantly improved the fruit quality. There are many indicators to evaluate the quality of fruit, and consumers prefer apples with a large fruit size, a full red color, and a suitable sugar–acid ratio. High firmness means better storage and transportation, but the application of Mg did not seem to change firmness in our study. The appropriate sugar–acid ratio can increase the flavor of apple, but the titratable acid content has no significant response to Mg, so the sugar–acid ratio is mainly regulated by the soluble sugar content in fruits. The key to the increase in soluble sugar content lies in the efficiency of synthesizing photosynthates and transporting to the fruits. In our study, Mg obviously promoted this process. We observed that Mg_150_ and Mg_200_ treatment promoted the synthesis of sucrose and sorbitol in leaves by improving photosynthetic efficiency and enzyme activity of SS, SPS, and S6PDH (**Table 2**; **Table 3**). Meanwhile, Mg promoted the transport of sorbitol and sucrose in the apoplastic pathway by upregulating the expression of *MdSOT1/3* and *MdSUT1/4* (**Figure 6**) and promoted the decomposition of sorbitol in fruits by increasing the activity of NAD^+^-SDH (**Table 3**), thus significantly increasing the contents of glucose and fructose (**Figure 5**). This not only effectively improves the sugar–acid ratio (**Table 1**) but also increases the pressure potential difference between the source and the sink by reducing the osmotic potential in the vacuoles, thus further promoting the transport of sorbitol through the symplastic pathway.

Scientific and effective N metabolism is conducive to the formation of fruit quality, and the lack of N will affect the synthesis and distribution of C assimilates and thus affect fruit quality. In our study, the application of Mg promoted the utilization and assimilation of N (**Figures 7A**; **8**), which enhanced the physiological and biochemical reactions and promoted the synthesis and transportation of photosynthate. The fruit expansion stage is the most critical period of fruit growth and development. Excessive N distribution in fruits reduces fruit quality (Wang et al., 2020a). However, our study showed that Mg application significantly improved the requisition and distribution of N in leaves and reduced the allocation of N to fruits (**Figures 2B**, **7B**). Therefore, appropriate application of Mg could also improve fruit quality by reducing N accumulation in fruits. Wang et al. (2020b) believed that less N distribution in fruits would also lead to reproductive growth restriction. In our study, appropriate application of Mg increased single fruit weight and fruit yield (**Table 1**), indicating that it did not lead to reproductive growth restriction. In fact, there is also an interaction between C and N metabolism. N metabolism promotes C metabolism by providing more photozyme and related pigments. Not only does C metabolism provides C skeleton for N metabolism but also products of C metabolism act as signaling substances to stimulate N uptake by plants (Peng et al., 2020b; Liu et al., 2022a). Therefore, the appropriate application of Mg can promote the C–N metabolism of trees and the C–N metabolism could promote each other and ultimately improve the fruit quality in common.

In addition to intrinsic quality, fruit color is also an economic trait. A higher anthocyanin content in peel is not only beneficial to apple skin coloring but also beneficial to human health. Wang et al. (2020b) showed that anthocyanins are synthesized through the flavonoid pathway and regulated by the expression of two types of genes, including structural genes encoding related synthases and transcription factors regulating structural genes, which included chalcone synthase (*MdCHS*), chalcone isomerase (*MdCHI*), flavanone 3-hydroxylase (*MdF3H*), dihydro flavonol reductase (*MdDFR*), and UDP-flavonoid dallyl transferase (*MdUFGT*), *MdMYB1* gene, and the *MdbZIP44* gene. Compared with CK treatment, Mg_150_ and Mg_200_ treatments could significantly upregulate the expression of *MdCHS*, *MdF3H*, *MdMYB1*, and *MdBZIP44*, and the anthocyanin content and fruit coloring were also improved (**Figure 1**).

Sugar is the precursor substance of anthocyanin synthesis, not only as an energy source but also as an osmoregulatory substance to promote anthocyanin synthesis through signal stimulation (Smeekens, 2000; Solfanelli et al., 2006). Loreti et al. (2008) showed that sucrose can upregulate the expression of related genes to promote anthocyanin biosynthesis in Arabidopsis. The contents of glucose and fructose in fruits were positively correlated with anthocyanin content (Huang et al., 2019). In addition, high N content in fruits would inhibit anthocyanin synthesis (Kühn et al., 2011). In our study, the application of Mg significantly decreased the concentration of N and significantly increased the concentration of glucose and fructose in fruits (**Figures 2B**, **5E, F**). Therefore, Mg_150_ and Mg_200_ treatments may regulate the expression of anthocyanin synthesis genes and transcription factors by changing the N signal and sugar signal, and the specific mechanism needs to be further studied.

## Conclusion

An appropriate application of Mg (150 kg/ha) increased NUE, promoted N assimilation, and decreased the allocation of N to fruits. Moreover, Mg promoted the synthesis of sucrose and sorbitol in leaves by improving the photosynthetic N use efficiency (PNUE) and enzyme activity of SS, SPS, and S6PDH. Meanwhile, Mg promoted the transport of sorbitol and sucrose to fruits by upregulating the expression of *MdSOT1/3* and *MdSUT1/4*. Subsequently, the decrease in N content and increase in sugar content in fruits may further stimulate the expression of anthocyanin synthesis genes to induce anthocyanin synthesis in the peel.

## Data availability statement

The original contributions presented in the study are included in the article/**Supplementary Material**. Further inquiries can be directed to the corresponding authors.

## Author contributions

SG and GT conceived and designed the experiments. GT and XX performed the experiments. CL, HQ and YX provided technical assistance. GT, JL, ML and ZF analyzed the data. GT wrote the manuscript. HJ, YJ and ZZ provided critical comments and revisions to the paper. All authors contributed to the article and approved the submitted version.
