# Supplementary material for: Magnesium improved fruit quality by regulating photosynthetic nitrogen use efficiency, carbon–nitrogen metabolism, and anthocyanin biosynthesis in ‘Red Fuji’ apple
Source: Front Plant Sci. 2023 Feb 23;14:1136179. doi: 10.3389/fpls.2023.1136179 (PMC9995890; doi:10.3389/fpls.2023.1136179)
Supplement: Supplementary file 1 [file Table_1.docx]

***Supplementary Material***

**Magnesium Improved Fruit Quality by Regulating** **Carbon-Nitrogen** **Metabolism and Anthocyanin Biosynthesis** **in 'Red Fuji' Apple**

**Ge Tian, Chunling Liu, Hanhan Qin, Yue Xing, Ziquan Feng, Xinxiang Xu, Jingquan Liu, Mengxue Lyu, Han Jiang, Zhanling Zhu*, Shunfeng Ge* and Yuanmao Jiang***

***Correspondence:**Shunfeng Ge [geshunfeng210@126.com](mailto:geshunfeng210@126.com)

Zhanling Zhu [zhlzh@sdau.edu.cn](mailto:zhlzh@sdau.edu.cn)

Yuanmao Jiang [ymjiang@sdau.edu.cn](mailto:ymjiang@sdau.edu.cn)

**Table S1** Primer sequences for PCR

| Gene | Forward primer sequence (5′→3′) | Reverse primer sequence (5′→3′) |
| --- | --- | --- |
| *MdACTIN* | TGGTGTCATGGTTGGTATGG | CCGTGCTCAATGGGATACTT |
| *MdCHS* | GGAGACAACTGGAGAAGGACTGGAA | CGACATTGATACTGGTGTCTTCA |
| *MdCHI* | GGGATAACCTCGCGGCCAAA | GCATCCATGCCGGAAGCTACAA |
| *MdF3H* | TGGAAGCTTGTGAGGACTGGGGT | CTCCTCCGATGGCAAATCAAAGA |
| *MdDFR* | GATAGGGTTTGAGTTCAAGTA | TCTCCTCAGCAGCCTCAGTTTTCT |
| *MdUFGT* | CCACCGCCCTTCCAAACACTCT | CACCCTTATGTTACGCGGCATGT |
| *MdMYB1* | TGCCTGGACTCGAGAGGAAGACA | CCTGTTTCCCAAAAGCCTGTGAA |
| *MdbZIP44* | AGCAGCACCTGGACGATCTGACG | GGTGAAGCATGTCGGCAGTGGCC |
| *MdNRT2.1* | TTGAGAGTTTGACAAGATAAACGCA | AACTGACGGGTGGGGAAATC |
| *MdNRT2.2* | TCGCTCTGGGGATCCATACT | TGCCTCGTGGAGTGTCATTT |
| *MdNRT2.4* | GCTGGGGGTTACATAGCAGTTAGG | CCATTAACCAGTCCAATAATCTTACCA |
| *MdNRT2.5* | TGTTGGTCCCACTTCTACCG | TGTCACCAGCAGTTCCCATC |
| *MdSUT1* | GTGGTAATTTACCGGCATTTGTCG | AAGCTAGAGGCCGTAGGGCAAG |
| *MdSUT2* | CAGATGGTTTCCTTTCTTGTTGAG | TGCTGATACATAGTGAGTGGAACCT |
| *MdSUT4* | TGATTTACCTCCAGTTGGCATTG | GCCAAGTCCCAAAGACTCAATTC |
| *MdSOT1* | GACAGAACCTCAGACTCCAAAG | TGACCAGAGACCTGAACGATA |
| *MdSOT2* | CGTATCCAACTATGCCTTCTCC | GACACCGACGGCAAGAATAA |
| *MdSOT3* | GCCGGTACTCTAAACATCTACTC | GAAGATGACTCCCGCAAGAA |
